# A high-quality de novo genome assembly for clapper rail (*Rallus crepitans*)

**DOI:** 10.1093/g3journal/jkad097

**Published:** 2023-05-02

**Authors:** Elisa C Elizondo, Brant C Faircloth, Robb T Brumfield, Subir B Shakya, Vincenzo A Ellis, Carl J Schmidt, Adrienne I Kovach, W Gregory Shriver

**Affiliations:** Department of Entomology and Wildlife Ecology, University of Delaware, Newark, DE 19716, USA; Museum of Natural Science and Department of Biological Sciences, Louisiana State University, Baton Rouge, LA 70803, USA; Museum of Natural Science and Department of Biological Sciences, Louisiana State University, Baton Rouge, LA 70803, USA; Museum of Natural Science and Department of Biological Sciences, Louisiana State University, Baton Rouge, LA 70803, USA; Department of Entomology and Wildlife Ecology, University of Delaware, Newark, DE 19716, USA; Department of Animal and Food Sciences, University of Delaware, Newark, DE 19716, USA; Department of Natural Resources, University of New Hampshire, Durham, NH 03824, USA; Department of Entomology and Wildlife Ecology, University of Delaware, Newark, DE 19716, USA

**Keywords:** Rallidae, reference genome, Hi-C Chicago, illumina shotgun

## Abstract

The clapper rail (*Rallus crepitans*), of the family Rallidae, is a secretive marsh bird species that is adapted for high salinity habitats. They are very similar in appearance to the closely related king rail (*R. elegans*), but while king rails are limited primarily to freshwater marshes, clapper rails are highly adapted to tolerate salt marshes. Both species can be found in brackish marshes where they freely hybridize, but the distribution of their respective habitats precludes the formation of a continuous hybrid zone and secondary contact can occur repeatedly. This system, thus, provides unique opportunities to investigate the underlying mechanisms driving their differential salinity tolerance as well as the maintenance of the species boundary between the 2 species. To facilitate these studies, we assembled a de novo reference genome assembly for a female clapper rail. Chicago and HiC libraries were prepared as input for the Dovetail HiRise pipeline to scaffold the genome. The pipeline, however, did not recover the Z chromosome so a custom script was used to assemble the Z chromosome. We generated a near chromosome level assembly with a total length of 994.8 Mb comprising 13,226 scaffolds. The assembly had a scaffold N50 was 82.7 Mb, L50 of four, and had a BUSCO completeness score of 92%. This assembly is among the most contiguous genomes among the species in the family Rallidae. It will serve as an important tool in future studies on avian salinity tolerance, interspecific hybridization, and speciation.

## Introduction

**Fig. 1.**
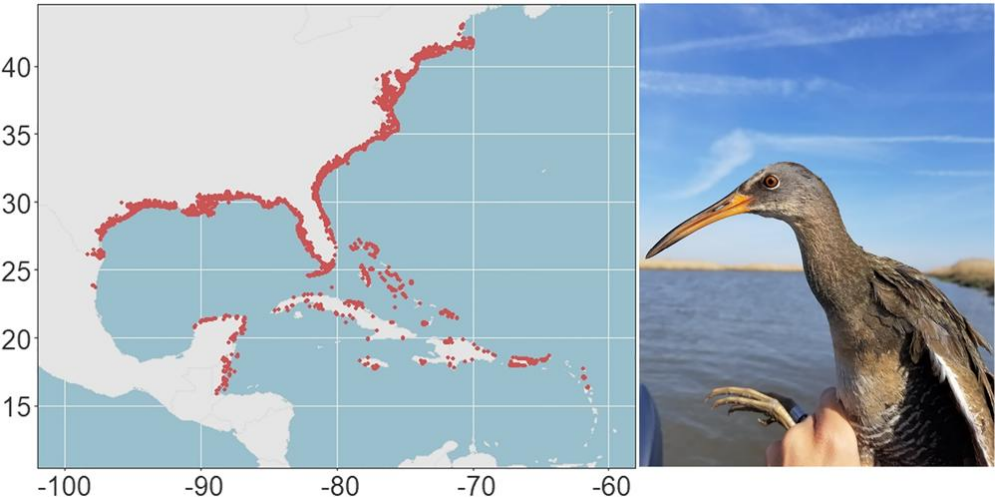
Clapper rail (*Rallus crepitans*) range produced using observational data from eBird (Sullivan *et al*. 2009) along with an image of a clapper rail captured in Woodland Beach, Delaware (photo credit: Elisa Elizondo).

Rallids (Aves: Rallidae) include 37 genera and 159 globally distributed species that occur primarily in wetlands, jungle lowlands, and montane forests (Garcia–R *et al*. 2019; Winkler *et al*. 2020). Despite their global distribution, most rallid species remain poorly understood because of their secretive nature. The type genus *Rallus* includes thirteen species of slim bodied, long-billed rails that occur in the Americas, Eurasia, Africa, and Madagascar (Winkler *et al*. 2020). Clapper rail (*Rallus crepitans*) and king rail (*R. elegans*) are 2 closely related species that occur along the eastern coast of North America, south to the Caribbean (Fig. 1; Rush *et al*. 2020). Clapper and king rails are similar in plumage, vocalization, and morphology (Maley and Brumfield 2013), but they exhibit different habitat preferences for saltwater (clapper rail) and freshwater (king rail) wetlands. The internal nasal salt glands of clapper rails are larger than those of king rails, and this adaptation is believed to contribute to the salinity tolerance (Conway *et al*. 1988) of clapper rails, although salt gland size is known to be a plastic trait that varies based on the water salinity to which the birds are exposed (Conway *et al*. 1988; Olson 1997). Osteologically, a narrower interorbital bridge in clapper rails accommodates its larger salt gland, and this species difference does not appear to be plastic, at least to the same extent as the salt gland (Olson 1997). Clapper and king rail populations hybridize where they cooccur in brackish marsh (Olson 1997; Maley 2012).

Avian hybridization along salinity gradients in North American marshes occurs not only between clapper and king rails but also between Nelson's sparrows (*Ammospiza nelsoni*) and saltmarsh sparrows (*Ammospiza caudacuta*) (Shriver *et al*. 2005; Walsh *et al*. 2016b, 2019). Similar to king rails, Nelson's sparrows are more closely associated with fresh and brackish wetlands, while saltmarsh sparrows, like clapper rails, are considered salt marsh obligates (Greenberg *et al*. 2006; Greenlaw *et al*. 2018). In the Nelson's/saltmarsh sparrow hybrid zone, genes associated with osmoregulation and salinity tolerance exhibit increased introgression, leading to improved fitness when hybrids are compared to Nelson's sparrows nesting in brackish and salt marshes (Walsh *et al*. 2016a). This observation suggests that for some organisms, hybridization may facilitate expansion into increasingly saline environments and additional work is warranted to explore these dynamics in other taxa. As climate change and sea level rise alter tidal marsh salinity gradients, it is increasingly important to understand how organisms can adapt to these changes in salinity.

To facilitate molecular investigations of the underlying mechanisms of saltwater tolerance and adaptive divergence between clapper and king rail populations, we completed the first genome assembly for clapper rail using DNA from a vouchered, wild female bird collected in Louisiana. To produce a chromosome-level assembly, we scaffolded contigs assembled using Meraculous (Chapman *et al*. 2011) and Spades (Bankevich *et al*. 2012) using Chicago and Hi-C libraries (Dovetail Genomics LLC). The resulting reference genome will be foundational to future studies investigating adaptation to high salinity environments, species limits in actively hybridizing populations, the conservation of *Rallus* species, and the genetic effects of sea level rise on marsh taxa.

## Methods

### Specimen collection and DNA extraction

Because we were interested generating data from both sex chromosomes, we collected a female clapper rail from Barataria Bay (saltwater), Plaquemines Parish, Louisiana (LSU IACUC 18-054; Louisiana Department of Wildlife and Fish Permit 18-022; US Fish and Wildlife Service Permit MB02467D); prepared a voucher specimen (Buckner *et al*. 2021) for the Louisiana State University Museum of Natural Science (LSUMNS) Collection of Birds (LSUMZ 199649); and archived muscle, liver, and other tissues in the LSUMNS Collection of Genetic Resources (LSUMZ B-95207). We shipped liver tissue to Dovetail Genomics, LLC (Scotts Valley, CA) where Dovetail staff performed high molecular weight (HMW) DNA extraction using the Blood and Cell Culture Midi Kit (Qiagen, Gmbh).

### Library preparation, sequencing, and assembly

Following HMW DNA extraction, Dovetail staff fragmented the DNA, prepared short insert sequencing libraries using an Illumina TruSeq DNA PCR-free kit, and sequenced the DNA using paired-end (PE) 150 base pair (BP) sequencing on an Illumina HiSeq X. The resulting data were trimmed to remove bases with quality scores lower than 20 using Trimmomatic (Bolger *et al*. 2014), and we used meryl 1.3 (k = 20; https://github.com/marbl/meryl) and Genomescope (Vurture *et al*. 2017) to estimate the genome size and heterozygosity of the sampled individual.

Dovetail staff used in-house software to profile the trimmed reads at a variety of k-mer values (19, 31, 49, 79, 109) and fit negative binomial models to the data to determine the best k-mer value for assembly. The constrained heterozygous model with 49-mers and a homozygous peak depth of 42.0 was selected as optimal for the assembly. Dovetail staff then assembled contigs using Meraculous with a k-mer value of 49, a minimum k-mer frequency of seven, and the diploid nonredundant haplotigs mode.

Following contig assembly, Dovetail staff used remaining tissue to prepare a single, proprietary “Chicago” library following the methods described in Putnam *et al*. (2016) and summarized in Salter *et al*. (2019). They sequenced the resulting Chicago library on an Illumina HiSeq X using PE, 150 bp reads to a depth of approximately 70X. Similarly, Dovetail staff prepared one HiC library from remaining tissue following the methods described in Lieberman-Aiden *et al*. (2009) and summarized in Salter *et al*. (2019). Dovetail staff sequenced the resulting HiC library to a depth of approximately 45X using PE, 150 bp reads on an Illumina HiSeq X. After preparing and sequencing Chicago and HiC libraries, Dovetail staff used HiRise (Putnam *et al.* 2016) to conduct two rounds of scaffolding: (1) using the Chicago reads to scaffold the Meraculous contigs and (2) using the HiC reads to scaffold the Chicago scaffolds. We refer to the resulting assembly as the “Dovetail HiC Assembly.”

After receiving the Dovetail HiC Assembly, we computed contiguity statistics using assembly-stats (https://github.com/sanger-pathogens/assembly-stats) and estimated assembly completeness using BUSCO v5.1.3 (Manni *et al*. 2021) and aves_odb10. While evaluating this version of the assembly, we noticed that the Z chromosome appeared to be missing. Specifically, after aligning scaffolds and contigs from the Dovetail HiC Assembly to the chicken genome assembly (UCSC galGal6; NCBI GCF_000002315.5) using ragtag v1.0.1 (Alonge *et al*. 2019), we did not recover any contigs or scaffolds that aligned to the chicken Z chromosome, suggesting Z chromosome contigs and scaffolds were not present. This problem has been observed in other Dovetail assemblies of birds (Del-Rio *et al*. 2021; Recuerda *et al*. 2021; Shakya *et al*. 2021) and may have resulted from the coverage parameters used by Dovetail during the Meraculous assembly process inadvertently excluding contigs representing sex chromosomes.

We addressed this problem by maintaining the macrochromosomes (scaffolds > 20 Mbp) from the Dovetail HiC Assembly while reassembling and rescaffolding contigs representing the microchromosomes. To start the microchromosome reassembly process, we trimmed the short-insert sequencing reads with trimmomatic v0.39 and corrected the trimmed reads using Musket v1.1 (Liu *et al*. 2013) and a kmer value of 61. We then performed a second de novo assembly using spades v3.14.0 (Andrey *et al*. 2020) with error correction turned off (−only-assembler) on a high-memory (1.5 TB) compute node, and we filtered the resulting assembly using faFilter (Kent *et al*. 2002) to remove contigs < 1 kbp. We extracted macrochromosomes (scaffolds > 20 Mbp) from the Dovetail HiC Assembly using faSize (Kent *et al*. 2002) and custom Python code, concatenated each into a single file, and used ragtag to align the contigs output by spades to this file macrochromosomes. Because of the way that ragtag formats output files, we were able to separate the contigs that aligned to macrochromosomes from those that did not, and we used custom Python code to create a file of contigs that did not align to the macrochromosomes. We provided this file of contigs to Dovetail staff, who reran the Chicago and HiC scaffolding processes using their proprietary HiRise pipeline.

After rescaffolding, we merged the resulting scaffolds (many representing microchromosomes) into the file of macrochromosomes to produce an assembly representing the entire genome, and we sorted the file by descending scaffold length using sortbyname in BBMap 38.78 (Bushnell 2014). We used custom Python code to rename all scaffolds, and we used faFilter to remove contigs/scaffolds shorter than 1000 bp in length. To ensure that the updated assembly contained scaffolds representing the Z chromosome, we performed a second alignment of the updated assembly to the chicken genome assembly (galGal6).

After validating that the updated assembly contained a large scaffold representing the Z chromosome, we used BWA v0.7.17 (Li 2013) to align reads from the short-insert libraries to the assembly, SAMtools v1.1.0 (Li *et al*. 2009) to sort and index the resulting BAM file, and Pilon 1.23 (Walker *et al*. 2014) to polish the assembly by fixing “–all” of the issues identified. We modeled repeats in the polished assembly using RepeatModeler v2.0.1 (Smith and Hubley 2008), and we soft-masked repeats using the output of RepeatMasker v 4.1.0 (Smith *et al*. 2013) with BedTools (Quinlan and Hall 2010). After polishing and repeat-masking, we checked the resulting assembly for adapter and other contamination using the NCBI Foreign Contamination Screen (FCS) tool (https://github.com/ncbi/fcs), we removed scaffolds/contigs that represented contamination, and we removed bases from scaffolds/contigs that matched adapter sequences. We also identified scaffolds/contigs that represented mitochondrial contamination by mapping the assembly to the mtDNA genome of *R. limicola* (CM040152.1) using minimap2 (v2.17-r941; Li 2018) and removing those scaffolds/contigs that matched (>90% length, >90% identity) portions of this mtDNA sequence. After making these changes, we sorted the remaining scaffolds/contigs by size and renamed them in order of decreasing length using custom Python code, and we used meryl 1.3 and Merqury 1.3 (Rhie *et al*. 2020) to compute reference-free estimates of k-mer completeness and consensus quality.

To produce a contact map of the resulting assembly, we removed adapters and low-quality bases from the HiC reads using trimmomatic, and we mapped trimmed reads to the assembly using BWA (v0.7.17) and SAMtools (v1.10). We used Picard (v.2.27.5; http://broadinstitute.github.io/picard) to sort and deduplicate properly aligned reads, and we produced a contact map of the deduplicated data using PretextMap (v0.1.9; https://github.com/wtsi-hpag/PretextMap) and PretextView (v0.2.5; https://github.com/wtsi-hpag/PretextView). We also assembled the mitochondrial genome by inputting trimmed reads from the short-insert libraries to MitoFinder v1.4.1 (Li *et al*. 2016; Allio *et al*. 2020) along with the NCBI reference sequence of *R. indicus* (NC_068741.1), which MitoFinder uses to identify mitochondrial reads during the initial stages of assembling a mitochondrial genome.

To ensure that repeat annotations exactly matched the names and coordinates in this final version of the assembly, we removed the soft-masking from the assembly, reran RepeatMasker with the repeat models we created, and soft-masked repeats using BEDTools. We assigned the Tree of Life Identifier (ToLID; https://id.tol.sanger.ac.uk) bRalCre1.1 to this version of the assembly, computed final set of contiguity statistics (assembly-stats) and BUSCO scores (aves_odb10) for this assembly version, and archived bRalCre1.1 with NCBI Genome. To compare bRalCre1.1 with genome assemblies from other rallids, we downloaded all assemblies for the family, and we computed contiguity statistics using assembly-stats and completeness estimates using BUSCO (aves_odb10) for each.

## Results and discussion

Short-insert library sequencing produced 325 million read pairs with an approximate insert size of 382 bp, and Genomescope results suggested that the *Rallus* genome was ∼1.3 Gb with a relatively low heterozygosity of 0.75 to 0.76%. Meraculous assembly using a k-mer value of 49 output 55,528 contigs with a total length of 990.8 Mb, a N50 of 50 kb (L50 = 5,380), and a maximum contig length of 606.9 kb (Table 1).

Chicago library sequencing produced 254 million read pairs, and HiRise made 27,838 joins and 24 breaks to the Meraculous assembly, producing an intermediate Chicago assembly including 19,218 scaffolds and having a total length of 994.3 Mb, a N50 of 1.8 Mb (L50 = 128), a N90 of 0.06 Mb (L90 = 1384), and a maximum scaffold length of 13.8 Mb. HiC library sequencing produced 170 million read pairs, and HiRise made 5,992 joins and zero breaks to the Chicago assembly. Fifty-seven gaps in the resulting assembly were closed using short-insert reads to produce the Dovetail HiC Assembly that included 13,226 scaffolds having a total length of 994.9 Mb, a N50 of 82.7 Mb (L50 = 4) scaffolds, a N90 of 10.8 Mb (L90 = 18), and a maximum scaffold length of 204 Mb. BUSCO completeness estimates for the Dovetail HiC Assembly are provided in Table 2.

**Table 1.** Contiguity statistics for *Rallus crepitans* assemblies comparing the Dovetail HiC Assembly and the bRalCre1.1 assembly.

	Dovetail HiC Assembly	bRalCre1.1
Scaffolds	13,226	12,159
Total length (Mb)	994.8	1,107.5
N50 (Mb)	82.7	82.9
N90 (Mb)	10.8	12.2
L50	4	4
L90	18	20
Longest scaffold (Mb)	204.0	204.6
# Ns	4,085,069	3,899,784
# Gaps	42,269	41,488

**Table 2.** Estimates of assembly completeness using the BUSCO aves_odb10 database (n = 8338 BUSCOs) showing the improvements in completeness between the Dovetail HiC Assembly and the bRalCre1.1 assembly, which includes the Z chromosome.

	Dovetail HiC Assembly		bRalCre1.1	
	Count	Percentage	Count	Percentage
Complete BUSCOs	7130	85.6	7671	92.0
Complete and single-copy BUSCOs	7117	85.4	7616	91.3
Complete and duplicated BUSCOs	13	0.2	55	0.7
Fragmented BUSCOs	314	3.8	216	2.6
Missing BUSCOs	894	10.6	451	5.4

Contig re-assembly using spades output 55,026 contigs having a total length of 1.1 Gb, a N50 of 58.0 kb (L50 = 4,904), a N90 of 9.5 kb (L90 = 22,795), and a maximum contig length of 907 kb. We identified 24,773 contigs that did not align to macrochromosomes in the Dovetail HiC assembly and we submitted these to Dovetail for re-scaffolding, which output a set of 12,193 scaffolds having an N50 of 15.3 Mb (L50 = 5) and a N90 of 8 Kb (L90 = 673). The longest scaffold in the re-assembly was 76.1 Mb in length and primarily aligned to the chicken Z chromosome. After merging the macrochromosomes from the Dovetail HiC Assembly with these scaffolds representing the microchromosomes and unplaced contigs and polishing the assembly, we removed 4 contigs identified by the NCBI FCS tools as alphaproteobacteria or eukaryotic viruses, masked 44 bases that corresponded to known adapter sequences, and removed 5 contigs identified as mitochondrial contamination. The contact map illustrated that HiRise performed well when scaffolding large (>100 kb) macro- and micro-chromosomes (Supplementary Fig. 1), although we could not discern a shift in the distribution of scaffold lengths that potentially differentiated microchromosomes from unplaced scaffolds (Supplemental Data). MitoFinder assembled a contig representing the mitochondrial genome that was similar in length (17.1 kb) to other rail species.

The final version of the assembly, bRalCre1.1, included 12,159 scaffolds/contigs having a total length of 1.1 Gb, a N50 of 82.9 Mb (L50 = 4), a N90 of 12.2 Mb (L90 = 20), and a maximum scaffold length of 204.6 Mb. BUSCO completeness estimates for bRalCre1.1 improved on the results from the Dovetail HiC Assembly (Table 2), although several BUSCOs remained fragmented (n = 216; 2.6%) or were not detected (n = 451; 5.4%). Merqury results suggested that bRalCre1.1 was relatively complete (kmer completeness = 91.4%) and accurate (consensus quality = 55.2 or > 99.999% accuracy). Repetitive elements comprised ∼9% of the assembly (Supplementary Table 2), and a majority of these repeats were retroelements.

The bRalCre1.1 assembly we produced is the second for a species in the genus *Rallus* and one of six assemblies representing taxa within the Rallidae. Our assembly is among the most contiguous for the taxonomic family (Supplementary Table 1), and the availability of a genome assembly representing this genus will facilitate investigations of salinity tolerance, interspecific hybridization, and mechanisms of speciation in clapper and king rails.

## Supplementary Material

jkad097_Supplementary_Data

## Data Availability

All short-insert, Chicago, and HiC sequencing data are available as part of NCBI BioProject PRJNA926626. The Whole Genome Shotgun project for bRalCre1.1 has been deposited at DDBJ/ENA/GenBank under the accession JAQOTC000000000. The version described in this paper is version JAQOTC010000000. Supplementary Table 1, Supplementary Fig. 1, a list of steps used to assemble the genome that includes the Python code used, Genomescope results, the PretextMap, Merqury results, RepeatMasker annotations, and results from BUSCO analyses of other rallid genomes are available from FigShare (https://doi.org/10.6084/m9.figshare.21983261).
