# Supplementary figures and images for: A high-quality de novo genome assembly for clapper rail (Rallus crepitans)
Source: G3 (Bethesda). 2023 May 2;13(8):jkad097. doi: 10.1093/g3journal/jkad097 (PMC10484055; doi:10.1093/g3journal/jkad097)

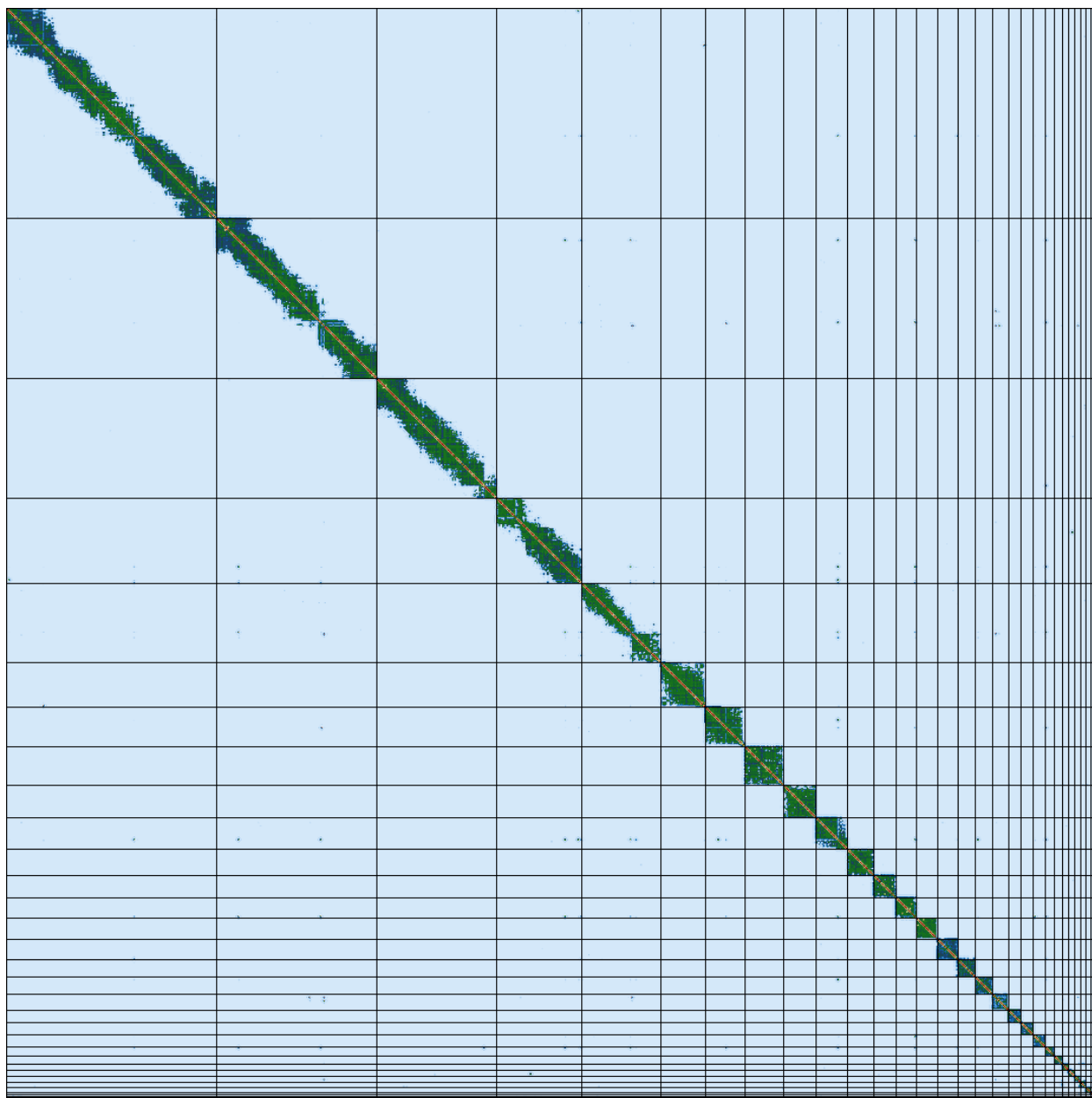

**Supplemental Figure 1.** Contact map of scaffolds  $\geq 100$  kb (sc1 to sc62) for bRaICre1.1, ordered by size.

Supplement: jkad097_Supplementary_Data [file jkad097_supplementary_data.zip › 1-Supplemental Figure 1.pdf]
